# Supportive and Rejective Functions of Tumor Stroma on Tumor Cell Growth, Survival, and Invasivity: The Cancer Evolution

**DOI:** 10.3389/fonc.2015.00044

**Published:** 2015-02-20

**Authors:** Jozsef Dudas

**Affiliations:** ^1^Department of Otorhinolaryngology, Medical University of Innsbruck, Innsbruck, Austria

**Keywords:** polarization, tumor microenvironment, immune cells, carcinoma-associated fibroblasts, stiffness

Tumors are complex organs composed of reciprocally interacting cell types including cancer-initiating cells, more or less differentiated cancer cells, extracellular matrix (ECM), and a variety of stromal cells such as endothelial cells, immune cells, pericytes, adipocytes, and fibroblasts (1). The tumor microenvironment (TME) comprises a variety of cell types lying among a network of various ECM fibers merged within the interstitial fluid and gradients of several chemical compounds, which constantly interplay with malignant cells (2). The TME is also energetically linked to cancer cells, which orchestrates functional changes in mitochondria of tumor cells that accompanies resistance to intrinsic apoptotic stimuli and contributes to hypoxia (3). The interaction of tumor resident cells impacts on the composition and texture of the matrix, including its stiffness. Tumor cells interacting with fibroblastic microenviroment might undergo a peculiar phenotypic change, known as epithelial–mesenchymal transition (2, 4, 5), and gain a pseudomesenchymal phenotype. These transdifferentiated cancer cells promote stiffening of their environment, which in turn feeds back in form of loss of tissue architecture or induction of cell invasion (2). Additionally, the ECM components shape the immune properties of dendritic cells (DCs) in the tumor (6), and finally contribute to an anergic state in these cells, favoring tumor immune-escape. Benencia et al. in this Research Topic discuss that this “immune paralysis” of cancer-associated DCs can be overcome in an experimental setting by blocking interleukin (IL)-10R while simultaneously activating specific pattern recognition receptors (6). In agreement, also Accolla et al. describes that the establishment of a pro-tumor environment is not the cause but simply the consequence of the tumor strategy to primarily counteract components of the adaptive cellular immunity, particularly T helper lymphocytes (7).

Stroma cells within the TME might show diverse polarization states, which is well-known in case of immune cells (8), but is a novel assumption in case of carcinoma-associated fibroblasts (CAFs) (1). Within the TME immune cells might destroy cancer cells, or they may promote tumor growth and dissemination, which is largely due to different polarization of them (8). Also, the CAFs have more populations, including the one, which is assigned with pro-tumorigenic effects as stimulating tumor growth and progression, and also the one, which might have tumor-inhibitory effects (1). The novel point of view is the acknowledgment of the plasticity and polarized phenotype both in the tumor cell populations and also in the microenvironment (1, 8). The polarized phenotypes and plasticity in both tumor cells and stroma enables a dynamic array of inhibitory and stimulatory signals in the tumor organ, which represents a new challenge for investigation of interactive processes instead of assuming cells with predefined roles. This is the basis of a continuous evolution of the tumor tissue.

According to contributions to this research topic (6, 7) the dynamism of TME might be influenced. The optimal initiation of the adaptive immune response dictated by adequate antigen availability, as evidenced by several research groups and reviewed by Accolla et al. is sufficient to reorient the TME from a pro-tumor to an anti-tumor one (7).

Similarly to immune cells, also fibroblasts might contribute to pro and to anti-TME. In an original article of Kaler et al. (9), authors report that normal intestinal fibroblasts activate STAT1 signaling in colon cancer cells and, in contrast to CAFs, inhibit growth of tumor cells (9). Interestingly, the proinflammatory cytokine TNFα could release this inhibition.

Detailed analysis of not only the potent mediators but also the signaling components is required in the stromal component of the tumor tissue in addition to tumor cells. Investigation of the signaling in stroma independently from tumor cells is a novel challenge, which is elegantly addressed by Wallace et al. They utilized two model systems: one where protein kinase C β (PKCβ) was deleted in both the epithelial and stromal compartments, and second, where PKCβ was deleted only in the stromal compartment (10). Mice globally lacking PKCβ were living longer and developed smaller tumors, and when PKCβ was exclusively lacking in the stroma, breast cancer tumor cells formed smaller tumors as well, with diminished collagen deposition. Interestingly, the stroma PKCβ-loss had no effect on tumor cell proliferation, vascularization, or macrophage infiltration (10). In summary, this research topic highlights the importance of the investigation and targeting of TME dynamism, and of understanding the stroma cell polarization.

## Conflict of Interest Statement

The author declares that the research was conducted in the absence of any commercial or financial relationships that could be construed as a potential conflict of interest.
